# Immune Checkpoint Inhibitor Therapy for Bone Metastases: Specific Microenvironment and Current Situation

**DOI:** 10.1155/2021/8970173

**Published:** 2021-11-28

**Authors:** Chang Liu, Miao Wang, Changli Xu, Bo Li, Juxiang Chen, Jianchun Chen, Zhiwei Wang

**Affiliations:** ^1^Department of Orthopedics, Changhai Hospital, Naval Medical University (Second Military Medical University), Shanghai 200433, China; ^2^Department of Orthopedics, The 900th Hospital of Joint Logistic Support Force, Fuzhou, Fujian Province 350025, China; ^3^Department of Neurosurgery, Changhai Hospital, Naval Medical University (Second Military Medical University), Shanghai 200433, China; ^4^Department of Orthopedics, The Third Affiliated Hospital, Naval Medical University (Second Military Medical University), Shanghai 201805, China

## Abstract

The treatment of bone metastases is a thorny issue. Immunotherapy may be one of the few hopes for patients with unresectable bone metastases. Immune checkpoint inhibitors are the most commonly used immunotherapy drugs currently. In this review, the characteristics and interaction of bone metastases and their immune microenvironment were systematically discussed, and the relevant research progress of the immunological mechanism of tumor bone metastasis was reviewed. On this basis, we expounded the clinical application of immune checkpoint inhibitors for bone metastasis of common tumors, including non-small-cell lung cancer, renal cell carcinoma, prostate cancer, melanoma, and breast cancer. Then, the deficiencies and limitations in current researches were summarized. In-depth basic research on bone metastases and optimization of clinical treatment is needed.

## 1. Introduction

Bone is the third most common metastasis site of several solid tumors, including lung cancer, kidney cancer, breast cancer, prostate cancer, and melanoma [1]. Sometimes, the bone metastases may even be diagnosed before the confirmation or after surgical removal of the primary tumor. The bone metastases are infrequently cured and usually indicate a poor prognosis. Bone metastases may also lead to skeletal-related events (SREs), such as severe pain, pathological fractures, spinal cord and nerve compression, and calcium and phosphate homeostasis changes, which reduce the patients' quality of life [1, 2].

However, it is difficult to carry out effective surgical treatment for bone metastases. The reasons include but are not limited to the following. (1) Bone metastases usually tend to occur in multiple sites of the body, and some of the metastases cannot be detected by conventional radiological methods, which leads to a difficulty in completely removing by surgery. (2) For the metastases which have not destroyed the cortex of bone, it is difficult to identify the location during the operation. (3) For the bone lesions which are close to blood vessels and nerves, the risk of surgery is high. (4) Sufficient surgical margin may be impossible to achieve for some bone metastases, which will result in recurrence. (5) For bone metastases patients in poor physical conditions, the surgery does not help prolong survival and even cannot be tolerated. Except for surgery, the currently conservative therapy for bone metastases includes radiotherapy, chemotherapy, targeted therapy, and immunotherapy.

In recent years, the rapid development of cancer immunotherapy has yielded high expectations, as it has extended survival time and improved the quality of life of patients [3]. In 2013, cancer immunotherapy was chosen as an “annual breakthrough” in the journal Science [4]. Blocking the immune checkpoints is currently one of the most studied and widely used methods in tumor immunotherapy. The immune checkpoints, such as programmed cell death protein 1 (PD-1), programmed cell death 1 ligand 1 (PD-L1), and cytotoxic T lymphocyte-associated protein 4 (CTLA-4), are the most important signal molecules that mediate the immune escape of tumors. In lymph nodes, CTLA-4 expressed by T cells is a homolog of CD28. CTLA-4 binds to CD80 (B7-1) or CD86 (B7-2) on antigen-presenting cells (such as dendritic cells) to regulate the activation of naive T cells and memory T cells. In the tumor microenvironment, the PD-L1 expressed by some tumor cells inhibits the immune response of T cells when it binds to PD-1, which promotes an immunosuppressive environment. Other immune checkpoints have been confirmed, including T cell immunoglobulin domain and mucin domain-3 (Tim-3), V-domain Ig suppressor of T cell activation (VISTA), lymphocyte-activation-gene-3 (LAG-3), indoleamine 2,3-dioxygenase (IDO), and killer-cell immunoglobulin-like receptors (KIRs) [5, 6].

The immune checkpoint inhibitors (ICIs) can block the above-mentioned signals that help tumor's immune escape so that the immune response would be reactivated and the tumors would be killed by the patient's immune system. Since the first CTLA-4 inhibitor ipilimumab was approved by the US Food and Drug Administration (FDA) for the treatment of metastatic melanoma in 2011, several PD-1 inhibitors (nivolumab, pembrolizumab) and PD-L1 inhibitors (atezolizumab, avelumab, and durvalumab) have been approved by the FDA for the treatment of up to 12 different types of advanced solid tumors: Hodgkin and non-Hodgkin lymphomas, melanoma, Merkel cell carcinoma, and liver, kidney, cervical, head and neck, lung, gastric, colorectal, and bladder cancers [7] (Table 1). Many patients have benefited from ICIs in clinical trials and practical applications [8]. For patients with bone metastases, especially those who have no conditions for surgery, ICIs may bring new hope.

However, as far as we know, there is currently no systematic review that concentrates on the therapeutic effects of ICIs on bone metastases. Bone, as an immune organ, has a special immune microenvironment. Osteoclasts and osteoblasts, which reside in the bone, are also likely to affect the progression of bone metastases. The exact mechanism of the occurrence and development of bone metastases is not fully understood [9]. In the clinical application of ICIs to bone metastases, there are many confusing as well as phenomena that are not consistent with expectations. Herein, we analyze the particularity of bone immunity and the therapeutic effects of ICIs on bone metastases, aiming to summarize the deficiencies of existing literature and find the directions for further researches.

## 2. The Special Immune Microenvironment of Bone and Bone Metastases

As an immune organ, bone has a different immune microenvironment from other organs, which makes bone metastases different from the primary tumor and other metastases. The development of bone metastases is often accompanied by bone destruction, which breaks the balance between osteoblasts and osteoclasts and causes changes in the immune microenvironment. The specific immune microenvironment in bone, as well as the bone metastases with immune heterogeneity, may affect the efficacy of immunotherapy in bone metastases, which has been revealed in clinical practice. In conclusion, an in-depth understanding of the immune microenvironment in bone is critical for improving the efficacy of tumor immunotherapy [10].

### 2.1. Unique Immune Microenvironment in Bone

We have observed that some tumors preferentially metastasize to bone, while others rarely metastasize to bone. It indicated that there may be an association between tumor type and the microenvironment of the metastasis site. As the metastatic should have evaded the immune surveillance in the bloodstream and target organ [11], there is a “seed and soil” hypothesis. The hypothesis postulates that the prone metastatic organs are the product of a favorable interaction between the metastatic tumor cells (“seeds”) and the microenvironment in the metastasis site (“soil”) [12]. Bone metastases will occur when the primary tumor cells are compatible with the bone microenvironment. Reasons for this preference include efficient delivery into the red marrow, chemokine gradients, lodgment in hematopoietic stem cell (HSC) niches, and the growth-promoting soil supplied by areas of bone remodeling [13–15]. Therefore, there exists a unique immune microenvironment in bone compared with other sites.

**Figure 1:**
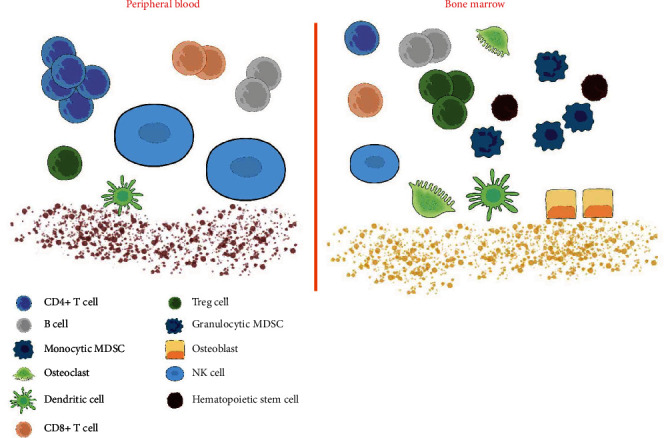
The difference between peripheral blood and bone marrow immune microenvironment. Compared with peripheral blood, there are a large number of immature and suppressive immune cell types in the bone marrow. CD4+ T cells, CD8+ T cells, NK cells, and other immune effector cells accounted for a small proportion, while immunosuppressive Treg cells and MDSCs accounted for a large proportion, which not only protected hematopoietic stem cell (HSC) but also weakened the immune killing effect on tumor cells. It provides an immune-privileged niche for disseminated tumor cells. A large number of immune factors are involved in the formation and regulation of osteoblasts and osteoclasts, which also affect the immune microenvironment of bone marrow.

Studies have shown that bone is a particularly immunocompromised area. The large amounts of immune cells in bone marrow seem to be unable to control the proliferation of cancer cells. This may be due to the existence of a large number of immature and inhibitory immune cell types in the bone niche, which weakens the activity of cytotoxic lymphocytes involved in tumor immune monitoring [13, 16]. In humans and mice, monocytes in peripheral blood are composed of 45-75% of T lymphocytes, including 25-60% of CD4+ and 5-30% of CD8+ T cells. However, in the bone marrow, the proportion of T cells decreased to less than 5% of monocytes, and CD8+ T cells were more abundant than CD4+ T cells [13, 17]. Similar to T cells, natural killer (NK) cells make up only 1-2% of lymphocytes in the bone marrow, although bone is the main site of their development [18] (Figure 1). In addition, NK cells play an ambiguous role in the bone niche. In several experiments of melanoma, prostate cancer, and breast cancer, they showed antitumor activity, while several data showed that NK cells promote melanoma proliferation and cancer stem cell (CSC) phenotypic transformation [16, 19]. There are also many noncytotoxic immune cells in bone, 40% of which are functional regulatory T cells (Tregs) [20]. Myeloid-derived suppressor cells (MDSCs) are other abundant immunosuppressive cells in bone marrow, which would expand during cancer progression and effectively inhibit CD4+ T cells, CD8+ T cells, and NK cells [21–24]. Moreover, the macrophages in the premetastatic niche contribute to the colonization of tumors that have metastasized to the bone [25]. In short, the bone marrow forms a unique immune cell compartment compared with other organs. It may provide an immune-privileged niche for disseminated tumor cells.

Osteoblasts and osteoclasts in bone are key components of bone homeostasis. Various immune cells and immune-related factors interact with osteoblasts and osteoclasts to achieve bone “balance” [26, 27]. Most of the factors involved in the differentiation of osteoclasts and preosteoclasts come from the immune system, such as macrophage colony-stimulating factor (M-CSF), interleukin (IL), transforming growth factor-*β* (TFG-*β*), prostaglandins, and interferon-*γ* (IFN-*γ*) [28]. T cells are also involved in the regulation of osteoclast activity [29, 30]. T_h_17 can induce the expression of M-CSF and RANKL (receptor activator of nuclear factor-kB ligand) in osteoblasts and stromal cells [31], produce RANKL and tumor necrosis factor-*α* (TNF-*α*), and simultaneously increase the expression of RANK in osteoclast precursors [32]. The migration of neutrophils can also recruit T_h_17, which can directly or indirectly induce the differentiation of osteoclasts [33]. Studies have shown that it is the Treg cells with positive CD4, CD25, and Foxp3 that suppress the differentiation of osteoclasts [34]. The Treg cells seem to suppress the differentiation of osteoclasts by inhibiting the release of TGF-*β* and IL-4, and IL-4 are CTLA-4 dependent [34], which is associated with current immunotherapies. In turn, osteoclasts also have immunomodulatory effects. The osteoclasts can present antigens, inhibit T cell initiation or proliferation, induce T cell incompetence, and express immunosuppressive and/or tolerable factors such as cytokines (including IL-10) and metabolic enzymes indoleamine 2,3-dioxygenase 1 (IDO1, which breaks down and depletes tryptophan, thus limiting T cell local activity) [35–38]. Wakkach et al. found that there was an inappropriate generation of B lymphocytes in mice lacking osteoclast activity, which resulted in reduced mature B cells and affected T cell activation, leading to the immunodeficiency of B-T cells [39]. For osteoblasts, although there are few immune-related studies at present, a large number of immune factors are found to be involved in their generation and regulation [28]. Meanwhile, osteoblasts, perivascular stromal cells, and endothelial cells are also components of the hematopoietic stem cell (HSC) niche and regulate the maintenance of HSC [40, 41]. The IL-7 and CXC chemokine ligand 12 (CXCL12), secreted by osteoblasts, are essential for B cell differentiation [42, 43].

In conclusion, there are a small number of effective cytotoxic cells and a relatively large number of immature or suppressive immune cells in the bone. There are also interactions among osteoclasts, osteoblasts, and immune cells. The unique immune microenvironment may provide an ideal site for hosting disseminated tumor cells and become a “refuge” for tumor cells to escape immunotherapy [13].

### 2.2. Correlation of Bone Metastases with Immunotherapy

On the one hand, the microenvironment in bone provides the “soil” for the colonized tumor cells (seeds). On the other hand, tumor cells can create an immune microenvironment suitable for proliferation at the metastatic site. Tumor cells release cytokines, which break the balance between osteoblasts and osteoclasts, to cause bone destruction and create an immunosuppressive microenvironment, thus promoting tumor progression and forming a “vicious cycle” [44–46]. As previously mentioned, increased osteoclast activity in patients with bone metastases is directly related to T cell involvement [29, 30]. In fact, enhanced osteoclast activity can be seen even in osteogenic metastases [47]. This interaction among tumor proliferation, bone destruction, and the immune response could be a basis for the treatment of bone metastases.

**Figure 2:**
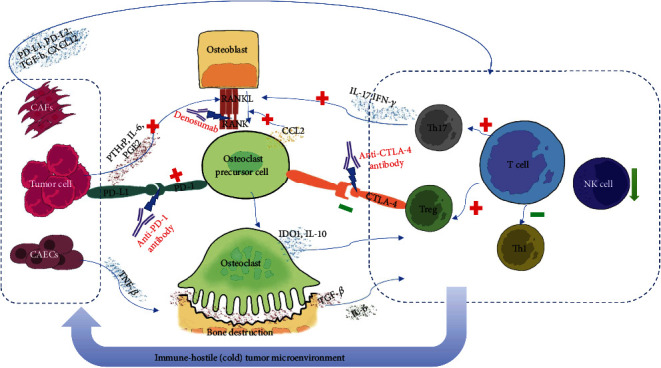
The interaction among the bone, immune system, and cancer cells. Tumor cells secrete PTHrP, PGE2, and other substances, which promote the transformation of osteoblasts into osteoclast precursors through the RANKL pathway and then differentiate into osteoclasts, causing bone destruction. Tumors can induce the release of CCl2 from the osteoclast precursors through the PD-1 pathway, which again promotes the occurrence of RANKL-induced osteoclasts. Osteoclasts secrete IDO-1, IL-10, and other substances to induce immunosuppression. TGF-*β* released by the destruction of bone and IL-6 in the microenvironment also causes immunosuppression. T cells differentiate into T_h_17 and Treg instead of T_h_1, forming an immune-hostile (cold) tumor microenvironment. T_h_17 secretes IL-17 and IFN-*γ* to promote osteoclast differentiation, while Treg cells rely on the CTLA-4 pathway to inhibit the transformation of osteoclast precursors to osteoclasts. PTHrP: parathyroid hormone-related peptide; PGE2: prostaglandin E2; CCI2: chemokine (C-C motif) ligand 2; IDO-1: indoleamine 2,3-dioxygenase 1; IL: interleukin; TGF-*β*: transforming growth factor-*β*; IFN-*γ*: interferon-*γ*.

A recent study revealed the increase of TGF-*β* in prostate bone metastases and the consequent changes in T cell differentiation. Jiao et al. [48] injected metastatic castration-resistant prostate cancer cells into the bone marrow of mice to establish a bone metastasis model of prostate cancer. The CTLA-4 inhibitor ipilimumab monotherapy did not induce an antitumor response in the model because of the deficiency of T_h_1 cell in the microenvironment. Further exploration indicated that the tumors in bone promote the bone resorption mediated by osteoclasts and release plentiful TGF-*β* into the microenvironment. Under the combined effect of TGF-*β* and IL-6 in the bone marrow, CD4+ T cells polarize to the T_h_17 cells rather than T_h_1 effector cells. Simultaneously blocking TGF-*β* and CTLA-4 (ipilimumab was used in the study) increased the number of CD4+ helper T cells in tumors, especially T_h_1 effector cells, leading to a significant increase in clonal amplification of CD8+ T cell and a decrease of Treg cells. The combination therapy significantly inhibited the progression of bone metastases and improved overall survival. However, the exact role of T_h_17 cells in anticancer immunity has not been verified. T_h_17 cells may be neither stimulatory like T_h_1 cells nor suppressive like Treg cells, but rather is an “inert” fraction [48]. As previously mentioned, cytokines secreted by CD4+ T_h_17 cells activate osteoclasts and promote bone resorption. It is suggested that this vicious cycle can also be blocked by immunotherapy [44, 45]. In addition, it is worth noting that previous studies have reported that Treg cells have CTLA-4-dependent antiosteoclast differentiation effect, and the blocking of CTLA-4 will reduce this effect of inhibiting osteoclast, which is worthy of further study [34] (Figure 2).

What is more, elevated TFG-*β* in bone metastases and its significance for ICI therapy have also been found in other studies. On the one hand, the role of TGF-*β* in promoting osteoclast differentiation is used by tumor cells to destroy the bone. On the other hand, as TGF-*β* is a key participant in inhibiting adaptive immunity, tumor cells in the bone can create an immune microenvironment with TGF-*β* to achieve immune escape and resistance to immunotherapy [49]. This suggests that adding anti-TGF-*β* therapy to the treatment of bone metastases may have good prospects. For example, preclinical studies in breast cancer and melanoma revealed that TGF-*β* promotes osteolytic metastasis of tumors [50–52]. Kang et al. analyzed 16 bone metastases samples from breast cancer, and 12 samples exhibited evidence of Smad pathway activation, which is a typical indicator of TGF-*β* stimulation [53]. As mentioned above, blocking TGF-*β* and CTLA-4 simultaneously in mouse models of castration-resistant prostate cancer with bone metastasis significantly inhibited the progression of bone metastasis and improved the overall survival rate compared with CTLA-4 inhibitor monotherapy [48]. In addition, downregulating the expression of TGF-*β* in tumors by oncolytic adenovirus targeting TGF-*β* demonstrated enhanced anti-PD-1 and anti-CTLA-4 therapeutic effects in animal models of kidney cancer and breast cancer [54].

Extracellular vesicles (EVs) secreted by tumors, which carry immunosuppressive molecules (such as PD-L1 and TGF-*β*), are also important mediators of tumor immune escape and possible targets for immunotherapy. The PD-L1 secreted by tumor-derived exosomes (TDEs) suppresses T cell activation in lymph nodes and promotes tumor proliferation at a distant site [55]. While anti-PD-L1 therapy is effective in reducing the immunosuppressive effect of cellular PD-L1, the effect on PD-L1 of TDE is poor, which explains why anti-PD-1/PD-L1 therapy is ineffective in some tumors with significant PD-L1 expression [56, 57]. Theodoraki et al. confirmed that PD-L1 expressed by TDEs in head and neck squamous cell carcinoma (HNSCC) patients was related to tumor development, lymph node involvement, and TNM staging [58]. Tucci et al. demonstrated that the PD-1 level of exosomes derived from DC and T lymphocytes can be used to predict overall and progression-free survival in melanoma populations [59]. EVs enriched in PD-L1 are of great significance to the prognosis and efficacy prediction of ICI treatment. Although we have not found a definitive study on the immunotherapy of EVs for bone metastasis, it seems that EVs and internal substances are likely to affect the efficacy of ICI therapy on bone metastasis.

Other studies have displayed PD-L1's promotion of osteoclastogenesis. In the study of Wang et al. [60], lung cancer cells were implanted into the femur of mice to make a femoral metastasis model. In this model, PD-L1 and chemokine (C-C motif) ligand 2 (CCl2) were upregulated, and PD-L1 induced osteoclastogenesis. PD-1 deficiency in mice avoided bone destruction during the development of intraosseous tumors. Nivolumab could inhibit the differentiation of tartrate-resistant acid phosphatase (TRAP)+ osteoclast in the femur, prevent bone destruction caused by bone metastases, and relieve pain caused by cancer in bone. However, nivolumab could not reduce the existing tumor burden in the bone. In mice lacking mature T cells, nivolumab still reduced bone destruction and pain, suggesting that nivolumab's protection against bone destruction does not require T cells but relies on its direct inhibition of osteoclastogenesis.

Bone metastases also change the microenvironment by interacting with nonimmune stromal cells in bone. The fibroblasts and endothelial cells are the two main stromal cell types. Fibroblasts, which are ubiquitous in normal bone marrow, can degrade and reshape the extracellular matrix, recruit immune cells, and promote angiogenesis after activation [61, 62]. Cancer-associated fibroblasts (CAFs) are fibroblasts that accumulate in tumors. The CAFs can form an immunosuppressive microenvironment through a variety of mechanisms (including upregulating the PD-1 expression of immune cells), which would promote tumor proliferation, cause tumor immune escape, and mediate drug resistance [63–65]. For example, CAFs conduce to the planting and growth of metastases in the bone by regulating the formation of the premetastasis niche [65]. In triple-negative breast cancer, the tumor subtypes rich in CAFs are more prone to bone metastasis, which is related to CXCL12 (C-X-C motif chemokine ligand 12) and IGF1 (insulin-like growth factor-1) cytokines and the CCL4-CCR5 (C-C motif chemokine ligand 4-C-C motif chemokine receptor 5) axis [66, 67]. In prostate cancer, the loss of TGF-*β* responsiveness in fibroblasts leads to the upregulation of CXCL16 and CXCL1 and promotes the growth of bone metastases [68]. Cancer-related endothelial cells also play a key role in immune escape and drug resistance of bone metastases. The increase in the density of blood vessels in bone promotes the colonization of tumors in the bone microenvironment [69]. Abnormal tumor blood vessels can hinder the delivery of immune cells and drugs and increase the motility of tumors [70].

The microenvironment of bone metastases in osteolytic and osteogenic prostate cancer is also different [71]. In osteolytic lesions, the immune infiltration of macrophages and T cells is increased, the PI3K-AKT (phosphatidylinositol 3 kinase-protein kinase B) signaling pathway is enhanced, and inflammatory drivers such as S100A8, S100A9, CCL5, and WNT5A are enriched. The B7-H3 immune checkpoint targets in T cell and macrophage compartments are upregulated. In osteogenic metastases, the immune cell infiltration is reduced, but tumor cells, macrophages, and T cells are rich for PD-L1 and IDO-1. Janus kinase-signal transducer and activator of transcription (JAK-STAT) signaling is enhanced. The expression of matrix metalloproteinase 7 (MMP7), laminin subunit gamma 2 (LAMC2), and SHC-transforming protein 2 (SHC2) is upregulated. It is suggested that targeted immunotherapy is necessary for different types of bone metastases.

In addition, the rapidly proliferating tumors in the bone can compete with immune cells for nutrition and produce immunosuppressive metabolites to induce immune cell failure and achieve immune escape [70].

### 2.3. Heterogeneity between Bone Metastases and Tumors in Other Sites

The metastases may already have different immunological characteristics from the primary lesions. It is well known that the immune system may transform tumors to be more aggressive and get stronger immune escape ability [72]. Cancer sequencing has also unraveled the genetic heterogeneity between primary and metastatic tumors caused by clonal evolution [73].

In prostate cancer patients, the differences in protein expression between bone and soft tissue metastases have been confirmed. Compared with soft tissue metastases, the expression of prosurvival proteins B cell lymphoma-2 (Bcl-2), myeloid cell leukaemia-1(MCL-1), and survivin-C is higher in bone metastases. However, the expression of prosurvival protein survivin-N is higher in soft tissue metastases [74].

By using bioinformatics techniques, Garcia-Mulero et al. found that the primary origin of bone metastases affects the immune phenotype of their subsequent metastases [12]. Compared with the primary lesions, bone metastases showed more abundance of stromal cells, enrichment in fibroblast, and significant differences in B lineage infiltration score [12]. They also discovered that metastases in different sites tend to be of different immunogenicity. Bone metastases tend to be medium immunogenic while lung metastases and liver metastases tend to be high and low immunogenic, respectively [12]. However, most bone metastases originating from colorectal cancer and kidney tumors belong to high immunogenic and show an increase in immune markers, which might be partly explained by the osteolytic nature of these lesions [12].

### 2.4. RANKL Pathway and Bone Metastases

The RANKL-RANK-OPG pathway consists of three components: receptor activator of nuclear factor-*κ*B ligand (RANKL), receptor activator of nuclear factor-*κ*B ligand (RANK), and osteoprotegerin (OPG) [75]. RANKL is expressed in two types: membrane bound and soluble. Membrane-bound RANKL mainly exerts the osteoclast effect [76]. In physiological conditions, RANKL produced by osteoblasts binds to RANK on the surface of the osteoclast precursor, which induces the osteoclast precursor to differentiate into multinucleated osteoclasts and activate osteoclasts to cause bone resorption. Then, osteoclasts produce a variety of factors that have nutritional effects on osteoblasts and promote the generation of new bone by osteoblasts. OPG is secreted by osteoblasts and osteoblastic stromal stem cells. OPG protects bone from excessive bone resorption by binding to RANKL and blocking the interaction between RANKL and RANK. Based on the mechanism of the RANKL-RANK-OPG pathway, denosumab, an inhibitor of RANKL, was approved by the FDA for severe osteoporosis, SRES, and nonresectable or metastatic giant cell tumors of bone and got promising results [77, 78].

Extracellular vesicles (EVs) are also involved in the regulation of bone balance by interacting with the RANKL pathway. It has been found that EVs from mature osteoclasts contained RANK, and the RANK in EVs may be associated with the inhibition of osteoclast formation [79]. This phenomenon may relate to the pathological bone imbalance causing by tumors. In multiple myeloma, a variety of molecules (including RANKL) carried by EVs promote osteolysis and inhibit osteoclast formation, which assists the tumor spread to distant bones [80, 81]. In the bone imbalance caused by bone metastasis of non-small-cell lung cancer, EVs also play roles by increasing the expression of RANKL in preosteoclasts [82].

The RANKL pathway plays an important role in bone metastases. The malignant cycle among tumor cells, bone marrow stromal cells, and osteoclasts, in which the RANKL pathway is the mediator, is the core mechanism of osteolytic metastasis. As mentioned above, osteoclasts, osteoblasts, and immune cells form a special immunosuppressive microenvironment in bone. Bone metastatic tumors produce various cytokines or factors, such as interleukin and TNF-*α*, to induce the expression of RANKL, leading to osteoclast activation, bone destruction, and immune resistance. RANKL is also expressed by immune cells such as NK and T cells [36, 83]. In the immune system, the RANK/OPG balance regulates the development of lymphocytes in lymph nodes, maintains the activation and maturation of dendritic cells, increases the production of immunosuppressive chemokines, and regulates the immune response mediated by T cells [84–86]. For osteogenic lesions, RANK and OPG also show correlations with bone metastases, even though the mechanism has not been confirmed [47]. The soluble RANKL can even exert chemotactic activity and promote tumor metastasis without osteoclasts [87].

In recent years, the RANKL pathway is considered as the link between bone and immune system, as well as targets for improving the efficacy of ICI therapy [16]. In the mouse model of bone metastasis of prostate cancer, the combination therapy of RANKL inhibitor and CTLA-4 inhibitor decreased the activity of osteoclasts and skewed bone remodeling toward osteoblastic activities [48]. In addition, blocking RANKL diminished TGF-*β*1 amounts in the tumor-bearing femur without affecting other cytokines [48]. Smyth et al. observed that the combination of anti-CTLA-4 and anti-RANKL demonstrated an antitumor activity in melanoma patients with bone metastases and found that the antitumor activity was dependent on lymphocytes in mouse model [88]. In mouse models, Ahern et al. confirmed that blocking RANKL improved the efficacy of not only CTLA-4 blockade but also PD1-PD-L1 blockade or dual PD1-PD-L1 and CTLA-4 blockade [89, 90]. The triple combination therapy of PD1-PD-L1 blockade, CTLA-4 blockade, and RANKL blockade further increased the proportion of tumor-infiltrating CD4+ and CD8+ T cells that can produce both IFN-*γ* and TNF [89]. A recent research in breast cancer revealed that the RANKL pathway is exploited by tumor cells to evade immune surveillance, and inhibiting RANK signaling increases the infiltration of leukocytes, lymphocytes, and CD8+ T cells [91].

## 3. Clinical Effects of Immune Checkpoint Inhibitors to Bone Metastasis

### 3.1. Non-Small-Cell Lung Cancer (NSCLC)

Immune checkpoint inhibitor (ICI) monotherapy or combined with chemotherapy has been approved as the standard treatment for advanced NSCLC, which accounts for 80% of lung cancer [92]. At present, the commonly used immune checkpoint inhibitors (ICIs) for NSCLC include the PD-1 inhibitor nivolumab and pembrolizumab and the PD-L1 inhibitor atezolizumab and durvalumab. The PD-1 and PD-L1 inhibitors are recommended for patients who progress after platinum-doublet chemotherapy, and pembrolizumab is approved for untreated patients with PD-L1 expression ≥ 50% [93]. Besides, due to the significant benefits in clinical research, dual immunotherapy (nivolumab+CTLA-4 inhibitor ipilimumab) has been approved by the FDA for the first-line treatment of PD-L1-positive (≥1%) and EGFR- or ALK-negative adult metastatic NSCLC in 2020 [94]. Nowadays, many clinical studies have demonstrated prolonged survival and improved quality of life for NSCLC patients who receive ICI treatment [3–6].

Bone is one of the most common metastatic sites of NSCLC [95, 96]. The incidence of bone metastasis in NSCLC patients is 20-40% [97–100]. Skeletal-related events (SREs) may occur in 30-60% of lung cancer patients with bone metastases [101]. Bone metastasis usually indicates a poor prognosis for patients with lung cancer [96, 97].

The prognostic significance of ICI therapy for NSCLC patients with bone metastases was not consistent, although a promising result was achieved in the general population in clinical practice. In many literatures, in line with most people's speculation, all prognostic indicators (such as progression-free survival, overall survival, and objective response rate) of patients with bone metastases significantly reduced whether the ICI was used alone or combined with other drugs [93, 98, 102–110]. However, different views are expressed. In Tamiya et al.'s study, no difference was observed in median progression-free survival (PFS) treated with nivolumab between advanced NSCLC patients with and without bone metastases [111]. A multicenter retrospective study of treating advanced NSCLC patients (PD-L1 tumor proportion score ≥ 50%) with pembrolizumab also indicated that there was no correlation between bone metastasis and PFS [100], which was opposite to another multicenter study in which the same type of patients was treated with the same ICI [105]. A nomogram to predict survival in NSCLC patients treated with nivolumab demonstrated that bone metastasis is not related to overall survival (OS) and PFS [99]. Interestingly, in Li et al.'s study of advanced NSCLC, the bone metastasis attenuated the efficacy of ICI monotherapy, and neither palliative radiotherapy nor bisphosphonates could improve the OS [112]. However, when ICI was combined with chemotherapy or antiangiogenic therapy, there was no difference in median PFS and OS between patients with and without bone metastasis [112].

Inconsistent outcomes of ICIs on bone lesions were also revealed, although only a few patients were involved in the current few studies and case reports [113, 114]. For NSCLC patients with bone metastases who were treated with nivolumab, 9 out of 12 patients developed metastatic progression in Schmid et al.'s study [115], while eight osteolytic lesions in 6 patients, out of 15 patients, had osteosclerotic changes in Nakata et al.'s study [116]. Contrary results also appeared in two case reports of nivolumab combined with radiotherapy in pleomorphic lung cancer patients. The bone metastases of Kodama et al.'s patient were well controlled [117], while the bone metastases of Kanazu et al.'s patient were still progressing during the treatment [118]. The difference between the two cases is that the primary tumor of Kodama et al.'s patient was resected surgically before treating with nivolumab. In another case report, the bone metastasis in the lower extremity and the primary lung tumor were significantly ameliorated in 2 patients treated with pembrolizumab [119]. Nakata et al. considered that assessing the early response of bone metastases with MD Anderson response classification criteria (MDA criteria) can be helpful to predict the prognosis of NSCLC patients with bone metastases [116]. The osteosclerotic change suggests the suppression of tumor and a better prognosis, while an increased number of bone metastases is associated with a higher risk of bone metastasis progression after nivolumab treatment [116].

We analyze that the following reasons may explain the inconsistent results above. The special immunosuppressive microenvironment in the bone [13, 113, 120] and the heterogeneity of bone metastases [121, 122], as we mentioned before, should be one of the important reasons. Besides, the target and dose of ICI and whether ICI is combined with other drugs may affect the results. In addition, mutations in genes or changes other than immune checkpoints in tumors may lead to a decreased efficacy of ICIs. For example, a change of epidermal growth factor receptor (EGFR) will make NSCLC more prone to bone metastasis [123] and resistance to ICIs [103, 123]. A concept of “hyperprogressive disease (HPD)” in NSCLC proposed by Choi et al. could be used to explain the different efficacy of ICI treatment in patients with bone metastases, as bone metastasis is one of the risk factors of HPD [124].

### 3.2. Renal Cell Carcinoma (RCC)

RCC originates from the renal tubular epithelium. Among all RCC patients, nearly 20-30% of patients are initially diagnosed as metastatic renal cell carcinoma (mRCC), and 20-40% of patients with localized tumors eventually develop into metastatic disease after surgery [125]. Bone is one of the most common metastatic sites of RCC. The incidence of bone metastasis in RCC is 30% [126]. The metastatic lesions are mainly osteolytic, which reduces bone integrity and causes skeletal-related events [127]. Bone metastasis is also one of the predictors of poor prognosis in mRCC patients [128–130].

The ICIs have brought the treatment of mRCC into a new stage. The commonly used ICIs for RCC include nivolumab and pembrolizumab for PD-1, atezolizumab and avelumab for PD-L1, and ipilimumab for CTLA-4. Both monotherapy and combination therapy have shown exciting therapeutic effects on advanced RCC [131–136]. The latest European RCC standards have upgraded immunotherapy to the first-line standard treatment options [137].

Similar to non-small-cell lung cancer, the prognostic significance of ICI therapy for RCC patients with bone metastases was inconsistent. In two recent studies, bone metastasis was independently associated with reduced PFS in patients with RCC treated with ICIs [103, 138]. However, in several real-world studies, there was no difference in FPS and objective response rate (ORR) between RCC patients with and without bone metastases [139–141]. Notably, as the subgroup analysis of CheckMate 025 clinical research demonstrated, ICIs actually increased the OS of patients with bone metastases from 13.8 months to 18.5 months [132]. The improvement of OS, PFS, and ORR was also observed when ICI was combined with tyrosine kinase inhibitor [133] or vascular endothelial growth factor receptor (VEGFR) inhibitor [134, 135] in some large multicenter studies, in which RCC patients with bone metastases accounted for a large proportion. Although appropriate subgroup analysis was insufficient in these multicenter studies, it seems that ICIs had a positive effect on RCC patients with bone metastases. For instance, in the clinical trial of Keynote 426 (24% of patients had bone metastases), the combination of pembrolizumab and tyrosine kinase inhibitor axitinib, compared with sunitinib monotherapy, improved the OS, PFS, and ORR both in the overall population and in subgroups with different risks regardless of the expression of PD-L1 [133].

The effect of ICI on the bone metastases of RCC is not clear. There is a case report that nivolumab monotherapy relieved bone metastases in a metastatic RCC patient who underwent radical nephrectomy [142]. Negishi et al. found that ICI monotherapy had limited effects on bone metastases, which may be related to the decrease of PD-L1 expression in bone metastases, while controlled bone metastases and reduced incidence of skeletal-related events emerged when using radiotherapy combined with ICI [143, 144]. A case report described the therapy of CT-guided percutaneous cryoablation combined with local administration of nivolumab to the primary tumor, which augmented the systemic immune response to elicit against bone metastases and made the uptake of metastatic bone lesions decrease in PET scan [145]. Based on the above phenomenon, ICI combined with other adjuvant therapy is promising for uncontrollable bone metastases.

In summary, although bone metastasis is a poor prognostic factor for RCC, the ICI provides hope for RCC patients with bone metastasis. For the bone metastases of RCC, ICI combined with other treatments seems to achieve better results than ICI monotherapy. It is worth noting that the reported detection rates of PD-1, PD-L1, and PD-L2 in RCC are 56.6%, 13.0-66.3%, and 21.0%, respectively, which is considered to be related to the metastasis and differentiation of cancer [146, 147]. The expression of immune checkpoints may be discrepant in each metastasis and the primary tumor [148]. The low expression of PD-L1 could be seen in bone metastasis compared with the primary lesion [147], which indicates that more targeted immunotherapy strategies are needed for RCC with bone metastasis.

### 3.3. Prostate Cancer

Prostate cancer is one of the most common cancers in men. Prostate cancer is relatively inert, and the survival rate of patients is relatively high. For unrestricted prostate cancer, radical mastectomy and chemical castration therapy are available treatments. However, some patients still suffer distant metastasis after castration therapy, and such a situation is called metastatic castration-resistant prostate cancer (mCRPC).

Bone is the most metastatic site of prostate cancer, and the bone metastasis incidence even reaches 90% in mCRPC patients [149]. As mentioned before, the balance of osteoblasts and osteoclasts in the bone metastases of prostate cancer is disturbed, which leads to tumor-induced bone disease (TIBD) and skeletal-related events. In the bone metastasis animal model of mCRPC, CD4+ T cells are polarized into the T_h_17 lineage instead of the T_h_1 lineage, and blocking TGF-*β* and CTLA-4 simultaneously can inhibit the progression of bone metastases [48]. The microenvironment of osteolytic and osteogenic metastases is also different [71].

It seems that ICIs have not achieved satisfactory effects in mCRPC patients [150–152]. For patients with bone metastases, the outcomes were even more disappointing. In a phase III clinical trial in patients with mCRPC treated with ipilimumab, patients with bone metastases had a worse response compared with patients with metastasis of other organs [152]. In the preliminary analysis of the CheckMate 650 Trial, when dual ICI therapy was received (ipilimumab combined with nivolumab), the PSA response rate was significantly lower in patients with bone metastases than in patients without bone metastases [153].

The unsatisfactory efficacy of ICIs may owe to the fact that prostate cancer is immunologically classified as a “cold tumor” which means immunologically ignorant, opposite to the “hot tumor” which means immune cell highly infiltrated tumor. Primary prostate cancer is characterized by low immune infiltration, low tumor mutation load, and low antigen presentation. The response to ICI monotherapy in prostate cancer is not as strong as that in non-small-cell lung cancer and renal cell carcinoma [8, 154]. In fact, the only immunotherapy that has been approved for prostate cancer is the Sipuleucel-T therapy of tumor vaccine, which has not achieved a satisfying outcome in mCRPC with bone metastasis yet [155, 156].

However, the possibility of precise treatment with a PD-1 inhibitor for mCRPC has emerged. It has been discovered that PD-1 inhibitor pembrolizumab may have a better effect on mCRPC patients with microsatellite instability high (MSI-H) [157]. The efficacy of CTLA-4 inhibitor ipilimumab combined with radiotherapy in mCRPC patients has also been assessed. Although no significant benefit of overall survival was presented, there were signs of decreasing risk ratio for overall survival over time, which warrant further investigation [150].

In brief, due to the “cold tumor” characteristics, ICIs are not ideal for treating primary prostate cancer recently, let alone bone metastases. Fortunately, Jiao et al.'s research [48] shows us the promise of ICI for prostate cancer. In addition, Ihle et al. considered that bone metastases should be classified as “hot tumor” [71]. His research team found increased immune infiltration and upregulated expression of immune checkpoint in the lytic specimens, as well as a higher level of PD-L1 and IDO-1 in blastic specimens [71]. Currently, a comparative trial of pembrolizumab for soft tissue and bone metastasis and a trial of monoclonal antibodies against B7-H3 are also ongoing or summarizing (NCT02628535, NCT02923180, and NCT01391143) [158]. There may be a breakthrough in the near future.

### 3.4. Melanoma

Malignant melanoma is highly aggressive and metastatic. About 1/3 of patients will have metastases, and the 5-year survival rate of patients with metastatic melanoma is less than 15% [159]. Although bone is relatively rare as the first metastasis site of melanoma, it is still the fourth most common metastasis site for melanoma, especially axial bone, with a reported rate ranging from 11 to 18% [160]. Bone metastases are also considered to be one of the poor prognostic factors in ICI therapy in advanced melanoma patients [160]. The bone metastases of melanoma are mostly osteolytic.

The history of immunotherapy for melanoma can be traced back to the approval of IL-2 therapy in 1998. However, the toxic side effects, low response rate, and low efficacy in improving survival rate have restricted its use. At present, the commonly used ICIs for melanoma are pembrolizumab and nivolumab for PD-1 and ipilimumab for CTLA-4. As the first CTLA-4 inhibitor approved in 2011, ipilimumab can effectively improve the patient's overall survival, but its immunological adverse reaction rate is still high [161]. Afterward, pembrolizumab and nivolumab have been approved for treating advanced melanoma due to their efficacy in longer overall survival time, higher progression-free survival rate, and lower incidence of adverse events [162, 163]. In addition, it seems that the combination of nivolumab and ipilimumab has achieved better results [164].

Surprisingly, we have not yet found subgroup analysis or real-world data in the current literature to clarify the impact of bone metastases in advanced melanoma treated by ICIs. Nor the trial that focused on the response of melanoma bone metastases to ICI monotherapy was found. To our knowledge, only a case report described an osteoblastic bone response and decreased lesions during treating with pembrolizumab [165]. The lack of literature is probably because bone is rarely the first metastatic site of melanoma, as most of the existing literature focused on the central nervous system and respiratory system metastasis.

However, some attempts to combine ICI with RANKL inhibitor to treat melanoma have been reported. In a case report, the combination of anti-CTLA-4 and anti-RANKL was used in a rapidly progressing metastatic melanoma patient with aggressive and symptomatic bone metastases, and no obvious residual lesion was found 62 weeks after diagnosis [88]. In two retrospective analyses of metastatic melanoma, no statistical difference was found in OS, PFS, and ORR between the ICI monotherapy group and the ICI combined with anti-RANKL group [166, 167]. However, in one of the two retrospective analyses, the combined therapy group contained more patients with bone metastasis and multiple metastases, which indicated a value of exploration in metastatic melanoma [166]. Angela et al. found that the change in melanoma bone metastases after combined therapy seemed to be consistent with the overall response of the tumor lesion in most cases, which means the consistent immune characteristics between bone metastases and the primary lesion [168].

Unlike renal cell carcinoma and prostate cancer, which have gained some experience in ICI combined with radiotherapy, the current research has not found the definite significance of this combined therapy in bone metastatic melanoma [169].

In summary, there is little literature on ICI therapy of bone metastatic melanoma, although ICIs have become the first-line treatment for unresectable or metastatic melanoma. A model of melanoma bone metastasis may be necessary to understand the effect of denosumab combined with ICI therapy. Several clinical trials exploring reasonable treatment options are also underway (such as NCT03161756, EudraCT No. 2016-001925-15). Some progress may appear soon.

### 3.5. Triple-Negative Breast Cancer (TNBC)

Bone is the most common metastasis site of breast cancer [170]. In a retrospective study of 18,322 breast cancer patients in China, bone metastases accounted for 39.8% of the total patients [171]. Luckily, among all patients with breast cancer metastasis, the survival rate of patients with bone metastasis is relatively higher [171].

The combination of atezolizumab and nab-paclitaxel treatment has been approved as the first-line treatment for unresectable or metastatic PD-L1-positive TNBC in 2019 [172]. The utility of anti-PD-1 for TNBC has also been demonstrated in clinical trials [173]. A phase 2 clinical trial on pembrolizumab combined with radiotherapy for TNBC also showed promising results [174].

Unfortunately, we have not found any suitable literature on the effect of ICI on breast cancer patients with bone metastasis and the bone lesion process. Actually, the positive rate of PD-L1 in different metastatic sites of TNBC is unequal, and it is only 16.7% in bone metastasis [172].

Luck in misfortune, promising results have been achieved in ICI combined with RANKL inhibitor therapy, which is similar to some cancers mentioned above. The RANKL/RANK axis is an important mediator of breast epithelial cell proliferation driven by progesterone and may contribute to the occurrence and development of breast cancer [175]. Breast tumor cells can secrete many different cytokines or factors, leading to an increase in RANKL produced by stromal cells and osteoblasts in the bone microenvironment [176]. Inhibiting the RANKL/RANK signaling pathway can transform the immune environment and enhance the efficacy of anti-CTLA-4 and anti-PD-1 treatment on breast cancer [175].

### 3.6. Other Carcinomas

In terms of urothelial cancer, anti-PD-1/PD-L1 has become the first-line treatment option [177]. A retrospective analysis of 270 patients in 10 European institutions treated with ICIs indicated that bone metastases were associated with poor prognosis [178]. In another study, 113 Asian patients with local progression or distant metastasis received PD-1 inhibitor tislelizumab treatment. The ORR was 24%, while the impact on bone metastasis was not specifically described [177].

ICI has not been successful in treating gastrointestinal solid malignant tumors, which implies that gastrointestinal tumors may employ other immunosuppressive mechanisms to affect the efficiency of immune responses [179]. The study on the combination of PD-1/PD-L1 targeting antibody and CXCL12-CXCR4/CXCR7 axis has been carried out in gastrointestinal malignancies [180]. In a case report of recurrent intrahepatic cholangiocarcinoma with bone metastasis, nivolumab combined with multitarget kinase inhibitor lenvatinib effectively inhibited the growth of bone metastases [181].

There are also some case reports demonstrating the therapeutic effect of ICI on bone metastases of some uncommon tumors. For example, in a sebaceous carcinoma patient with multiple organ metastases (including bone), pembrolizumab combined with radiotherapy suppressed the growth of metastases [182]. Coincidentally, in a malignant pleural mesothelioma patient with multiple metastases, complete response was observed in bone metastases after treating with pembrolizumab [183]. However, in a glioblastoma patient with metastatic dissemination who received nivolumab treatment, the bone metastases progressed while the brain tumors resolved, which may be the result of different immune microenvironments [184].

## 4. The Deficiencies and Limitations in Current Researches

As illustrated before, the treatment outcomes of the immune checkpoint inhibitors (ICIs) achieved in the actual clinical application did not seem to be as expected. Although gratifying results have been achieved in a small number of patients with bone metastases, the impact of ICIs on the prognosis of patients with bone metastases is still controversial. In many clinical researches, patients with bone metastases tend to have a worse prognosis. The efficacy of ICIs on bone metastases is often not ideal even if the PD-L1 expression in the primary tumor is high. Although some achievements have been made in the exploration of the bone microenvironment and the characteristics of bone metastases, we believe that there are still some deficiencies and limitations in current researches.

### 4.1. Inadequate Understanding of the Specificity of Immune Checkpoints in Bone Metastases

As mentioned in the relevant section of this review, the bone microenvironment is different from other target organs, and bone metastases are also heterogeneous with primary tumors and metastases in other sites. Only by fully understanding the peculiarities of treatment in bone metastases will the ICI therapy be more effective.

First of all, we need to realize that there are a relatively large number of immature or suppressive immune cells in the bone, which makes bone a site with low immune function. Intraosseous tumors also release factors to achieve immune escape. The special immune microenvironment in the bone creates objective conditions for tumor immune escape and may limit the activity of ICIs [13, 113]. Moreover, the molecular mechanism of tumor immune escape is very complicated, including but not limited to the PD-1/PD-L1 pathway. Tumors may choose other alternative molecules to achieve immune escape when the PD-1 pathway is blocked [5]. In such a complex tumor microenvironment, it may be difficult to achieve satisfactory outcomes using immune checkpoint inhibitors alone.

Secondly, as bone metastases are more heterogeneous than primary tumors and metastases in other organs, it may not be possible to predict the therapeutic effects of ICIs by the expression of PD-L1 alone. More and more evidence revealed that the expression of PD-L1 is dynamic [185]. The expression of PD-L1 may be mutative in different areas of the same tumor and at different time points of the disease, namely, spatial heterogeneity and temporal heterogeneity [121, 186]. Some tumors will show a decrease in PD-L1 expression following ICI treatment [121]. The expression of PD-L1 in bone lesions may be lower than in other lesions [121, 122]. In clinical practice, those patients with low or undetectable PD-L1 expression still showed clinical benefit during ICI treatment, which reveals that it is not reliable to guide ICI strategy through the expression of PD-L1 in biopsy or postoperative specimens [5, 9]. Therefore, other reliable biomarkers are urgently needed.

Thirdly, resistance to ICIs has been described in many literatures, including primary resistance and acquired resistance. Primary resistance refers to those cases in which no initial response to immune checkpoint inhibitor was observed. Acquired resistance encompasses cases in which the patients initially responded to an ICI but later became refractory [187]. The researches on the mechanism of ICI resistance may help improve the therapeutic effects.

### 4.2. Insufficient Assessment Methods for Bone Metastases

In the descriptions of the ICIs' therapeutic effects on bone metastases that we have found, the response of the lesions is often judged by the size, number, nature (osteolysis or osteogenesis), and 18F-FDG intake. However, in the era of targeted therapy and immune checkpoint inhibitors, these evaluation criteria may not reflect the true tumor response and cannot prove the actual clinical benefit [9].

Firstly, unlike traditional chemotherapy, targeted drugs may attract inflammatory cells to the tumor microenvironment, often causing angiogenic edema or intratumor hemorrhage. As a result, the lesions' diameter may increase on radiological examination at the initial stage of treatment, which would cause misunderstanding [188]. Secondly, it is inaccurate to classify bone lesions as either osteolytic or osteogenic. Osteolytic and osteogenic components coexist in most bone lesions [9]. Although using MDA criteria to evaluate the effect of nivolumab in bone metastases has been explored [112], there is no consensus on the best response evaluation system for bone-related lesions so far [9].

### 4.3. Inconsistent Standards and Deficiencies in the Evaluation Criteria in Clinical Researches

The characteristics of patients are diverse in the clinical practice. Therefore, apart from case reports, large-scale clinical trials and retrospective analysis based on the real world cannot ensure the homogeneity of patients, nor can they ensure the standardization of research and treatment methods. In the clinical studies we found, the evaluation of the effect of ICI treatment on patients with bone metastases was limited to indicators such as event-free survival rate, objective response rate, and overall survival rate. There is no description of the details we are concerned about, such as the combined treatment strategy, the number of bone metastases, the nature and location of bone metastases, and the progress and outcome of bone metastases. The inconsistent standards and deficiencies in the evaluation criteria affect the comparison between studies, as well as make it impossible to draw conclusions about whether bone metastasis is the factor for the poor prognosis in ICI treatment.

As we have learned, many factors of bone metastases may affect the efficacy of ICIs such as the location, nature, and number. Whether the primary lesion is resected and whether RANKL inhibitors or other drugs are used in conjunction also cause different results. For example, in a study on non-small-cell lung cancer patients with bone metastases, ICI monotherapy and ICI combined with other drugs led to different prognoses [112]. In a case report mentioned before, a good result was achieved for bone metastases in a patient who received ICI treatment after the removal of the primary tumor in the lung [117]. Therefore, it will be very meaningful to carry out researches on the effects of ICIs to bone metastases. We may obtain more valuable conclusions if we include whether the primary tumor has been surgically removed, the characteristics of bone metastases, and the metastases in other organs into the evaluation indicators. The targeted researches will be more helpful to provide references in ICI treatment for patients who have found bone metastases after the removal of the primary tumor or before the initial diagnosis.

## 5. Conclusion and Perspective

In conclusion, bone metastases are intractable. For patients with inoperable bone metastases, immunotherapy may be one of the limited hopes. However, bone is a special immune site with a unique immunosuppressive microenvironment. Tumors that are able to seed in bone may be heterogeneous and can even affect the intraosseous immune microenvironment in turn. Osteoblasts, osteoclasts, stromal cells, and the RANKL/RANK/OPG pathways make the microenvironment more complicated. On this basis, the clinical application of ICIs for bone metastasis is summarized. The prognostic effect of ICIs on non-small-cell lung cancer or renal cell carcinoma patients with bone metastasis, as well as the therapeutic effects on metastases, was inconsistent. For the bone metastasis of prostate cancer, the efficacy of ICIs was unsatisfactory. In melanoma or triple-negative breast cancer patients with bone metastases, the combination of ICI with RANKL inhibitor brought hope. Strengthening the understanding of the peculiarities in bone metastases, improving assessment methods, and designing specific researches will help to improve the therapeutic effect of ICIs on bone metastases.

It is noteworthy to conduct researches in the ICI therapy to bone metastases. As combined utilization of multiple types of ICIs [189], as well as ICI combined with RANKL inhibitor [43, 88] or VEGF inhibitor [112, 181], has achieved promising outcomes, multidrug combination may help prevent the immune escape of intraosseous tumors. There are still several clinical studies being conducted (Table 2). Of note, benefits have emerged when ICI was combined with radiotherapy [150, 182]. The sensitizing effect of radiotherapy on ICI therapy may be attributed to the radiation-enhanced steps that are required to generate an antigen-specific immune response, including the death of inflammatory tumor cells, activation of dendritic cell, cross-presentation of antigen, and activation and proliferation of cytotoxic T cell [190]. In addition, the abscopal effect, which describes the phenomenon that the tumors in the distant unirradiated area shrink during radiotherapy, may be improvable in ICI therapy as it has been displayed in several case reports [145, 191]. Furthermore, developing nanomaterials to assemble ICIs, which may make the ICI more targeting to bone metastases, is another direction and has been attempted [192]. Although the extracellular vesicles' effects of immunosuppression and bone balance regulation are being emphasized, we have not found a study on extracellular vesicles for the ICI therapy to bone metastasis, which calls for further exploration. Finding new biomarkers to predict the therapeutic outcomes of ICI to bone metastases will provide references to preoperative neoadjuvant therapy and postoperative treatment for advanced cancer patients.

## Figures and Tables

**Table 1:** FDA-approved immune checkpoint blocking antibodies.

Target	Approval drug	Indication
PD-1	Pembrolizumab	Melanoma, lung cancer (NSCLC, SCLC), head and neck squamous cell carcinoma (HNSCC), classical Hodgkin's lymphoma (CHL), primary mediastinal large B cell lymphoma (PMLBCL), urothelial carcinoma (UC), MSI-H cancer, gastric cancer, cervical cancer, hepatocellular carcinoma (HCC), Merkel cell carcinoma (MCC), renal cell carcinoma(RCC), endometrial carcinoma, bladder cancer
	Nivolumab	Melanoma, lung cancer (NSCLC, SCLC), renal cancer, classical Hodgkin's lymphoma, head and neck squamous cell tumor, urothelial carcinoma, MSI-H or dMMR metastatic colorectal cancer, HCC
	Cemiplimab	Squamous cell carcinoma of the skin

PD-L1	Atezolizumab	Urothelial carcinoma, lung cancer (NSCLC, SCLC), breast cancer
	Durvalumab	Urothelial carcinoma, NSCLC
	Avelumab	Merkel cell carcinoma (MCC), urothelial carcinoma

CTLA-4	Ipilimumab	Melanoma, renal cell carcinoma (RCC)

Abbreviations: MSI-H: microsatellite highly unstable; dMMR: mismatch-repair deficient; NSCLC: non-small-cell lung cancer; SCLC: small cell lung cancer.

**Table 2:** Ongoing clinical trials with ICIs in bone metastases.

Cancer	Trial identifier	Status	Intervention	Enrolment target (*n*)	Primary outcome
Non-small-cell lung cancer	NCT03996473	Active, not recruiting	Radium-223+pembrolizumab	164	Objective response rate (ORR)
	NCT03669523	Recruiting	Denosumab+nivolumab	86	Overall response rate (ORR), disease-control rate
Melanoma	NCT03161756	Recruiting	Ipilimumab, denosumab, nivolumab	72	Median progression-free survival, occurrence of grade 3 and 4 selected immune-related adverse events (irAEs) of interest
Unresectable or metastatic B7-H3-expressing neoplasms	NCT02628535	Terminated	B7-H3 × CD3 DART protein	67	Number of participants with adverse events
Renal cell carcinoma and urothelial carcinoma	NCT03291028	Recruiting	Immune checkpoint inhibitor targeting PD-1	16	Genomic and histopathological characterization of samples from ICB-treated patients
Prostate cancer	NCT03406858	Recruiting	HER2Bi-armed activated T cells and pembrolizumab	33	Progression-free survival
Breast cancer	NCT04841148	Recruiting	Hydroxychloroquine or avelumab, with or without palbociclib	96	Proportion of subjects in each treatment arm with clearance of DTC (disseminated tumor cells)
Melanoma	NCT04516122 ^∗^	Not yet recruiting	Nivolumab and pembrolizumab	40	Changes in bone density, change in done turnover markers

^∗^This trail focuses on bone destruction caused by ICI in the treatment of melanoma.
